# Understanding the molecular basis of agonist/antagonist mechanism of human mu opioid receptor through gaussian accelerated molecular dynamics method

**DOI:** 10.1038/s41598-017-08224-2

**Published:** 2017-08-10

**Authors:** Yeng-Tseng Wang, Yang-Hsiang Chan

**Affiliations:** 1 Department of Biochemistry, College of Medicine, Kaohsiung Medical University, Kaohsiung, Taiwan; 2 Center for Biomarkers and Biotech Drugs, Kaohsiung Medical University, Kaohsiung, Taiwan; 3 Graduate Institute of Medicine, Kaohsiung Medical University, Kaohsiung, Taiwan; 4 Department of Medical Research, Kaohsiung Medical University Hospital, Kaohsiung, Taiwan; 5 Department of Chemistry, National Sun Yat-sen University, 70 Lien Hai Road, Kaohsiung, Taiwan

## Abstract

The most powerful analgesic and addictive properties of opiate alkaloids are mediated by the μ opioid receptor (MOR). The MOR has been extensively investigated as a drug target in the twentieth century, with numerous compounds of varying efficacy being identified. We employed molecular dynamics and Gaussian accelerated molecular dynamics techniques to identify the binding mechanisms of MORs to BU72 (agonist) and β-funaltrexamine (antagonist). Our approach theoretically suggests that the 34 residues (Lys209–Phe221 and Ile301–Cys321) of the MORs were the key regions enabling the two compounds to bind to the active site of the MORs. When the MORs were in the holo form, the key region was in the open conformation. When the MORs were in the apo form, the key region was in the closed conformation. The key region might be responsible for the selectivity of new MOR agonists and antagonists.

## Introduction

Opioid receptors are G protein-coupled receptors (GPCRs) and are potential drug targets utilized for pain relief and the treatment of pain-related disorders. Since thousands of years, opiates (morphine) have been used to relieve pain resulting from numerous disorders, particularly diarrhea, acute pain, and cancer pain. The opioid system plays a critical role in the modulation of pain behavior and antinociception^1^. Opioid-related proteins are expressed throughout the nociceptive neural circuitry in the central nervous system; this circuitry is associated with reward- and emotion-related brain structures^2^. The four types of opioid receptors [mu (μ), delta (δ), kappa (κ), and opioid receptor like-1] have been characterized at protein, cellular, molecular, and pharmacological levels^3^. The most powerful analgesic and addictive properties of opiate alkaloids are mediated by the μ opioid receptor (MOR)^4^. Activation of the MOR results in signaling through the heterotrimeric G protein, resulting in sedation and analgesia. The MOR can also mediate signaling through arrestin, and this pathway contributes to the adverse effects of opioid analgesics including antinociceptive tolerance, physical dependence, respiratory suppression, and constipation^5, 6^. The MOR has been extensively investigated as a drug target in the twentieth century, with numerous compounds of varying efficacy being identified. Because of the serious side effects of morphine, scientists have made considerable progress in the development of new opioids^1^.

GPCRs are cell transmembrane receptors that play fundamental roles in pathophysiology and physiology by mediating cellular responses to various agonists including peptides, hormones, photons, odorants, amines, proteins, nucleotides, and lipids^7^. Most GPCRs have been suggested to exist in an ensemble of different conformational states (inactive, ligand-free, and active)^8^. The conformation of GPCRs is biased toward the active state when bound by agonists. By contrast, GPCRs switch to the inactive state upon binding of antagonists^9^. In addition, their conformation is biased toward the ligand-free state when not bound by agonists or antagonists. Moreover, identifying the ligand-free states of GPCRs can facilitate the development of more selective drugs capable of modulating a specific signaling pathway, thereby minimizing undesirable side effects and improving therapeutic efficacy^10, 11^. The X-ray structure of the MOR has been determined in the active state, in which the MOR is bound to the morphinan agonist BU72^12^. Currently, X-ray studies have revealed the inactive structure of the MOR^13^. Furthermore, the X-ray structures of the inactive/active states of the MOR have been obtained^12, 13^; however, because of the lack of experimental conformation of the MOR, many problems remain unresolved.

Studying the binding mechanisms of agonists and antagonists to GPCRs is difficult because long-time scale all-atom dynamics simulations are necessary for sampling conformational states of GPCRs^14, 15^. The application of all-atom molecular dynamics (MD) simulations for studying conformational ensembles obtained from a single, long-time-scale conventional molecular dynamics (cMD) simulation is still limited; this limitation is due to the possible energy barriers between various ligand-free states. Thus, an enhanced sampling technique is required for this task. Enhanced sampling techniques have been applied to predict the structural dynamics of GPCRs^16–21^. Recent reports show that enhanced sampling techniques have been successfully applied for evaluating binding mechanisms and structural dynamics^17^, including the metadynamics method^22^, adaptive biasing force^23^ method and coarse-grained conformational sampling, cMD^14^, and accelerated molecular dynamics (aMD) or Gaussian accelerated molecular dynamics (GaMD)^18^. These enhanced sampling studies provide crucial insights into binding mechanisms and structural dynamics. The disadvantage of enhanced sampling techniques is the requirement of predefined parameters (i.e., root-mean-square distance and protein structures). However, the enhanced sampling method of aMD (or GaMD) avoids such a requirement. In the aMD method, a boost potential is added into the potential energy surface; thus, the energy barriers are effectively decreased, accelerating transitions between the low-energy states^18, 24, 25^. The aMD method has also been successfully applied to biological system simulations, and aMD simulations conducted on the time scale of hundreds of nanoseconds can approach cMD simulations conducted on the millisecond timescale^26–29^.

The drawback of the aMD method is the large energetic noise occurring during reweighting^30^. In aMD simulations, the applied boost potential is typically on the order of tens to hundreds of kilocalories per mole (kcal/mol), which is much higher than that of other enhanced sampling methods that utilize protein structures or reaction coordinates. Accurately reweighting aMD simulations is difficult, particularly for large protein molecules^31^. Miao *et al*. provided a solution (i.e., GaMD) for improving the aMD method. In the GaMD method, the boost potential follows a near-Gaussian distribution, and cumulant expansion to the second order provides improved reweighting of aMD simulations^32^. The reweighted free energy profiles of GaMD are in good agreement with those of the long-time-scale cMD simulations^33^. However, GaMD has the limitation that it cannot evaluate proteins with less than approximately 35 amino acid residues^33^.

In this study, we applied the GaMD method to simulate the binding mechanisms of agonists and antagonists to a MOR and observed the structural dynamics of the MOR.

## Results and Discussion

### Free energy calculation (PMF) of complex MORs by using GaMD simulations

**Figure 1:**
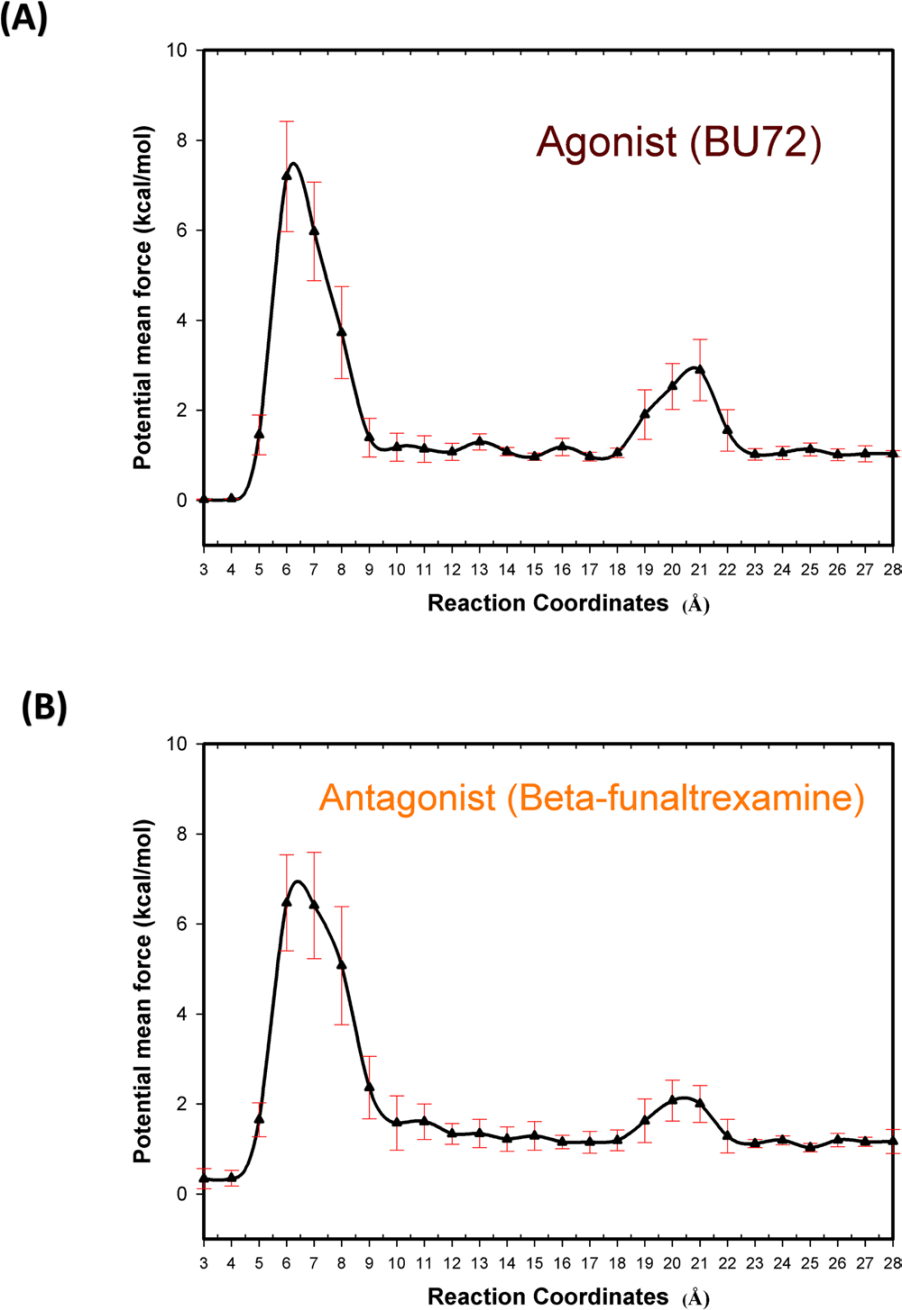
Free energy profiles (PMF) of reaction coordinates. The PMF profiles were calculated with five individual 1000-ns GaMD simulations. (**A**) Agonist (BU72) (**B**) Antagonist (Beta-funaltrexamine).

Free energy (PMF) profiles of complex systems were explored using GaMD simulations of MOR distance values, and the profiles are illustrated in Fig. 1. Snapshots of MORs with agonist (BU72) and antagonist (β-funaltrexamine) ligands are presented in Figures S1 and S2. Our PMF calculations indicate the presence of two energy barriers (major barrier: at RCs of 4–12 Å; minor barrier: at RCs of 18–23 Å) in the five independent 1000-ns GaMD simulations. For the MOR with agonist (BU72), the major energy barrier was 7.19 ± 1.22 kcal/mol and the minor barrier was 2.89 ± 0.68 kcal/mol. For the MOR with antagonist (β-funaltrexamine), the major energy barrier was 6.46 ± 1.06 kcal/mol and the minor barrier was 2.07 ± 0.45 kcal/mol. Moreover the energy barriers were subjected to RMSF calculations, and the snapshots of RCs (3 and 18 Å) were subjected to functionally key residue analysis.

### Functionally key residues

**Figure 2:**
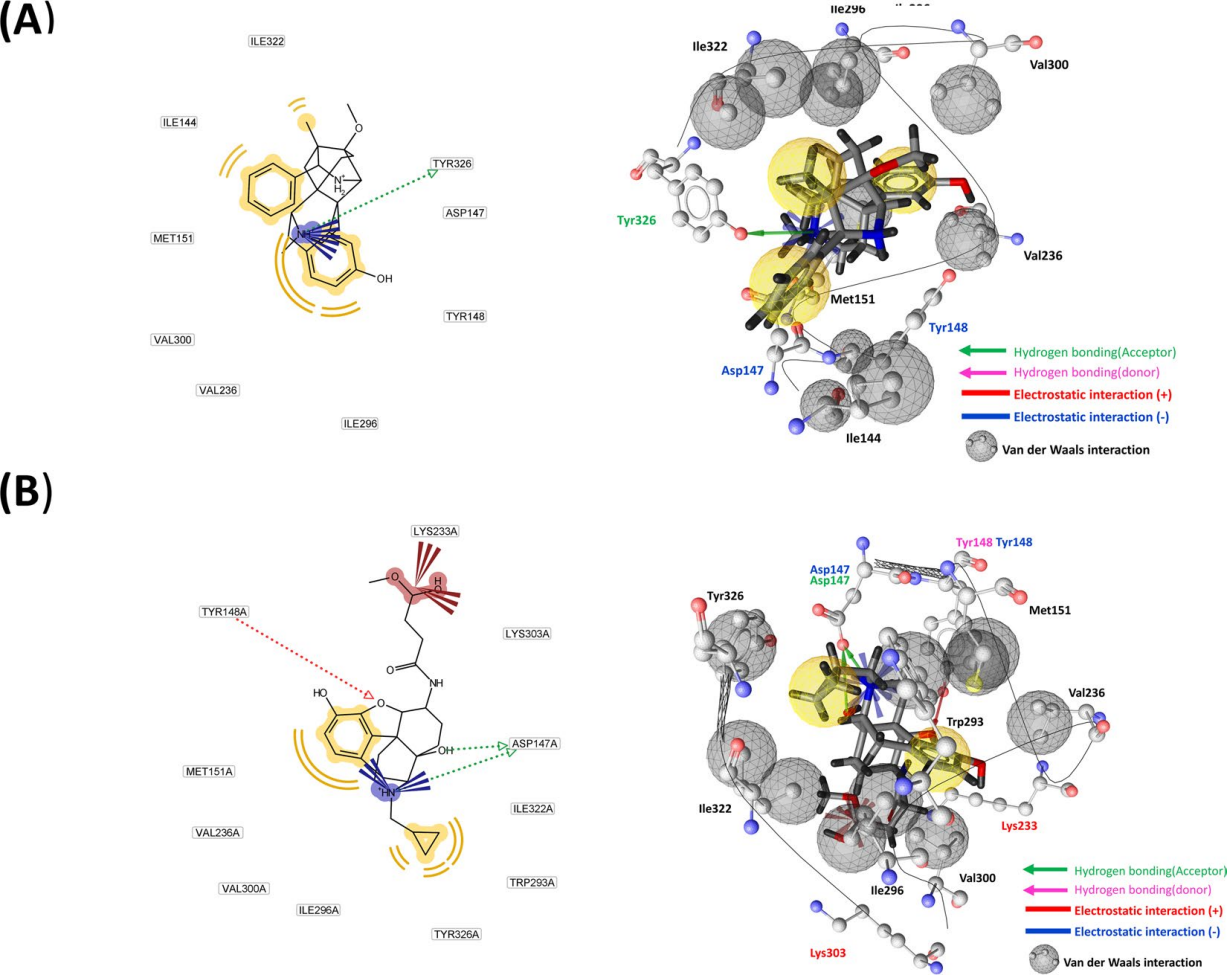
Binding modes (X-ray structures) of active/inactive MORs with BU72 and β-funaltrexamine. (**A**) Active MOR with BU72. (**B**) Inactive MOR with β-funaltrexamine.

Identification of functionally key residues can provide a clear insight into the structural aspects of MORs. In this study, a structure-based approach was applied to identify functionally key residues. According to the snapshots of the RC (18 Å) and the X-ray structures of the MORs, the key residues and pharmacophore regions were analyzed using the Ligandscout program. About the snapshots of the RC (18 Å), the residues (probability that more than half) were selected for the binding mode analysis. Our results are presented in Table 1 Fig. 2.

**Table 1:** Analysis of the binding modes of MORs.

Compounds	BU72	β-funaltrexamine
**X-ray structures of MORs (RC = 3 Å)**		
Electrostatic	Tyr148 and Asp147	Asp147, Tyr148, Lys233 and Lys303
Van der Waals	Ile322, Ile144, Val236, Met151, Val300 and Ile296	Val236, Val300, Met151, Ile296, Ile322, Trp293, Tyr326
Hydrogen bonding	Tyr326	Asp147 and Tyr148
*The snapshots of RC (18 Å)		
Electrostatic	Thr132 (52%), Ser214 (61%), Asp216 (86%) and Gly217 (57%)	Gln124 (51%), Asn127 (84%), Tyr128 (77%), Met130 (51%) and Asp216 (56%)
Van der Waals	Gly131 (62%), Thr132 (72%), Gly213 (51%) and Ile215 (62%)	Met65 (86%), Val66 (65%), Thr67 (88%), Ala68 (89%), Ile71 (79%), Tyr128 (84%) and Leu129 (62%)
Hydrogen bonding	Asp216 (54%)	Gln124 (63%), Asn127 (67%), Tyr128 (84%), Thr132 (52%) and Asp216 (53%)

^*^More than half chance: (RC 18 Å).

For the binding modes (X-ray structure) of BU72, two residues (Tyr97 and Asp96) exhibited frequent electrostatic interactions with BU72; six residues (Ile271, Ile93, Val185, Met100, Val249, and Ile245) exhibited frequent van der Waals interactions with BU72, and one residue (Tyr275) formed one hydrogen bond with BU72. For the binding modes (X-ray structure) of β-funaltrexamine, three residues (Tyr97, Asp96, and Lys252) exhibited frequent electrostatic interactions with β-funaltrexamine and seven residues (Met100, Val249, Ile245, Val185, Tyr275, Ile271, and Trp242) exhibited frequent van der Waals interactions with β-funaltrexamine.

For the binding modes (snapshots at an RC of 18 Å) of BU72, four residues (Thr132, Ser214, Asp216, and Cys217) exhibited frequent electrostatic interactions with BU72, four residues (Gly131, Thr132, Gly213, and Ile215) exhibited frequent van der Waals interactions with BU72, and one residue (Asp216) formed one hydrogen bond with BU72. For the binding modes (snapshots of RCs at 18 Å) of β-funaltrexamine, five residues (Gln124, Asn127, Tyr128, Met130, and Asp216) exhibited frequent electrostatic interactions with β-funaltrexamine, seven residues (Met65, Val66, Thr67, Ala68, Ile71, Tyr128, and Leu129) exhibited frequent van der Waals interactions with β-funaltrexamine, and five residue (Gln124, Asn127, Tyr128, Thr132, and Asp216) formed hydrogen bonds with β-funaltrexamine. For the binding modes (X-ray structure), the residues of Asp147, Tyr148, Val300, Ile296, Ile322, and Tyr326 interacted with BU72 and β-funaltrexamine simultaneously. For the binding modes (snapshots at an RC of 18 Å), the residues of Thr132 and Asp216 interacted with BU72 and β-funaltrexamine simultaneously. The results from the analysis of functionally key residues reveal that two compounds might exhibit different mechanisms for binding to MORs.

### MORs at an RC of 28 Å

Identifying the apo forms of MORs can provide a clear three-dimensional structure of MORs for designing drugs. Figure S3 shows two apo forms of MORs (at an RC of 28 Å). The RMSD between the two MOR apo forms was 2.05 Å. Figures S3(C) and S3(D) present a comparison of two X-ray structures of MORs with the apo forms of MORs.

### Electrostatic and van der Waals binding interactions (major barrier: at RCs of 4–12 Å; minor barrier: at RCs of 18–23 Å)

Table 2 shows the electrostatic/van der Waals binding interactions between key residues (Table 1) and the two compounds. For the MOR with agonist (BU72), the binding interactions were quickly decayed within the RCs of 5–7 and 19–21 Å. For the MOR with antagonist (β-funaltrexamine), the binding interactions were quickly decayed within the RCs of 4–7 and 19–21 Å.

**Table 2:** Analysis of the electrostatic and van der Waals binding interactions (major barrier: at RCs of 4–12 Å; minor barrier: at RCs of 18–23 Å).

RCs (Å)	Electrostatic interactions (kcal/mol)	van der Waal interactions (kcal/mol)
**BU72**		
4	−9.16 ± 1.51	−4.24 ± 1.16
5	−8.23 ± 1.10	−3.76 ± 1.21
6	−5.61 ± 1.19	−2.09 ± 1.18
7	−5.47 ± 1.05	−1.19 ± 1.04
8	−3.96 ± 1.35	−0.84 ± 0.41
9	−3.17 ± 0.61	−0.78 ± 0.04
10	−2.01 ± 0.37	−0.56 ± 0.03
18	−16.43 ± 1.34	−3.93 ± 1.21
19	−15.91 ± 1.04	−3.74 ± 1.01
20	−12.07 ± 1.61	−2.64 ± 1.94
21	−11.46 ± 1.14	−1.97 ± 1.37
22	−9.71 ± 1.64	−0.94 ± 0.09
23	−8.10 ± 1.51	−0.17 ± 0.06
**β-funaltrexamine**		
4	−12.74 ± 1.86	−5.91 ± 1.64
5	−9.18 ± 1.91	−2.37 ± 1.17
6	−9.04 ± 1.72	−2.91 ± 1.31
7	−8.69 ± 0.15	−2.14 ± 1.96
8	−6.71 ± 0.54	−1.84 ± 0.54
9	−6.08 ± 0.37	−0.94 ± 0.37
10	−5.49 ± 0.39	−0.87 ± 0.19
18	−17.15 ± 1.08	−5.41 ± 1.07
19	−16.75 ± 1.17	−4.91 ± 1.13
20	−12.07 ± 1.84	−2.06 ± 1.14
21	−11.36 ± 1.41	−1.74 ± 1.09
22	−9.54 ± 0.95	−0.94 ± 0.18
23	−8.42 ± 0.83	−0.77 ± 0.12

^*^The electrostatic/van der Waals binding interactions between key residues (Table 1) and the two compounds were conducted for two barriers (at RCs of 4–10 and 18–23 Å).

### Binding mechanism of BU72 (agonist) to MORs

**Figure 3:**
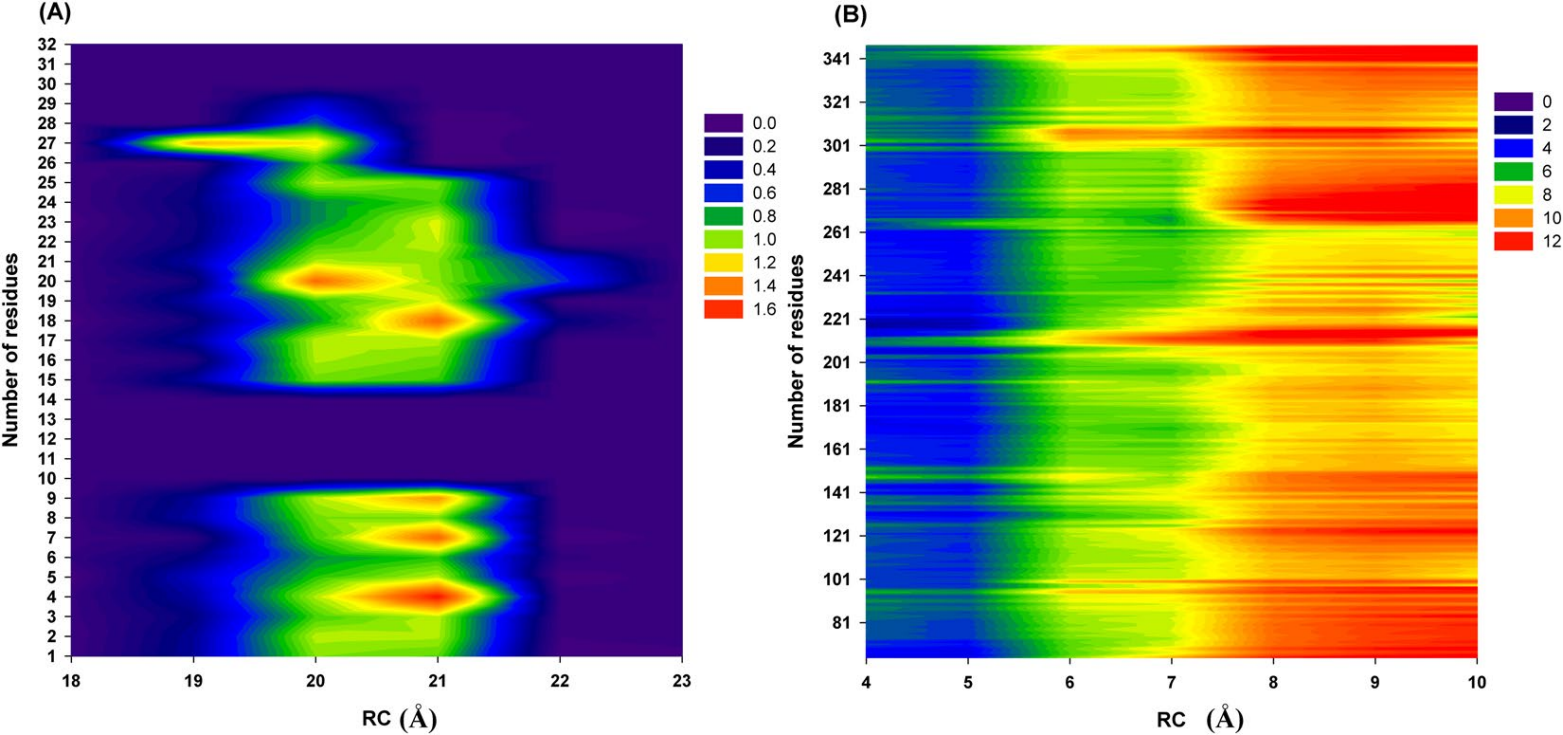
RMSF profiles of an MOR with BU72. (**A**) RC: 18–23 Å; the order of the residues is as follows: Val126, Asn127, Tyr128, Leu129, Met130, Gly131, Thr132, Trp133, Pro134, Cys140, Val143, Ile144, Asp147, Lys209, Tyr210, Arg211, Gln212, Gly213, Ser214, Ile215, Asp216, Cys217, Thr218, Leu219, Thr225, Trp226, Glu229, Lys303, Ala304, Leu305, Thr307, and Glu310. (**B**) RC: 3–10 Å.

As revealed in our PMF profiles, BU72 must overcome the two energy barriers (major barrier: at RCs of 4–14 Å; minor barrier: at RCs of 18–23 Å) to bind with the binding pocket (Table 2) of MORs. The possible residues interacting with BU72 in the minor barrier (at RCs of 18–23 Å) are presented Figs 1 and 3 as well as Table 1. First, BU72 must overcome the minor energy barrier (2.89 ± 0.68 kcal/mol). At RCs of 19–21 Å, the Val126, Asn127, Tyr128, Leu129, Met130, Gly131, Thr132, Trp133, Pro134, Tyr210, Arg211, Gln212, Gly213, Ser214, Ile215, Asp216, Cys217, Thr218, Leu219, Thr225, Trp226, and Glu229 residues (order: 1–9 and 15–27; RMSF > 1.00 Å) exhibited obvious fluctuations, particularly the Leu129, Thr132, Pro134, Gly213, and Ile215 residues (order: 4, 7, 9, 18, and 20). Table 2 also showed that the binding interactions were quickly decayed within the RCs of 19–21 Å. Our results showed that the residues might play important roles in relaxing Mors and making BU72 easy to overcome the minor energy barrier. Subsequently, BU72 must overcome the major energy barrier (7.19 ± 1.22 kcal/mol). At RCs of 7–11 Å, all residues exhibited obvious fluctuations (RMSF > 8 Å). At 5–7 Å, the Met90–Lys100, Lys209–Phe221, Ile301–Cys321, and Glu341-Phe347 residues exhibited obvious fluctuations (RMSF > 8 Å). Table 2 showed that the binding interactions were quickly decayed within the RCs of 5–7 Å. Our results showed that the Met90–Lys100, Lys209–Phe221, Ile301–Cys321, and Glu341-Phe347 residues might play important roles in relaxing Mors and making BU72 easy to overcome the major energy barrier.

### Binding mechanism of β-funaltrexamine (antagonist) to MORs

**Figure 4:**
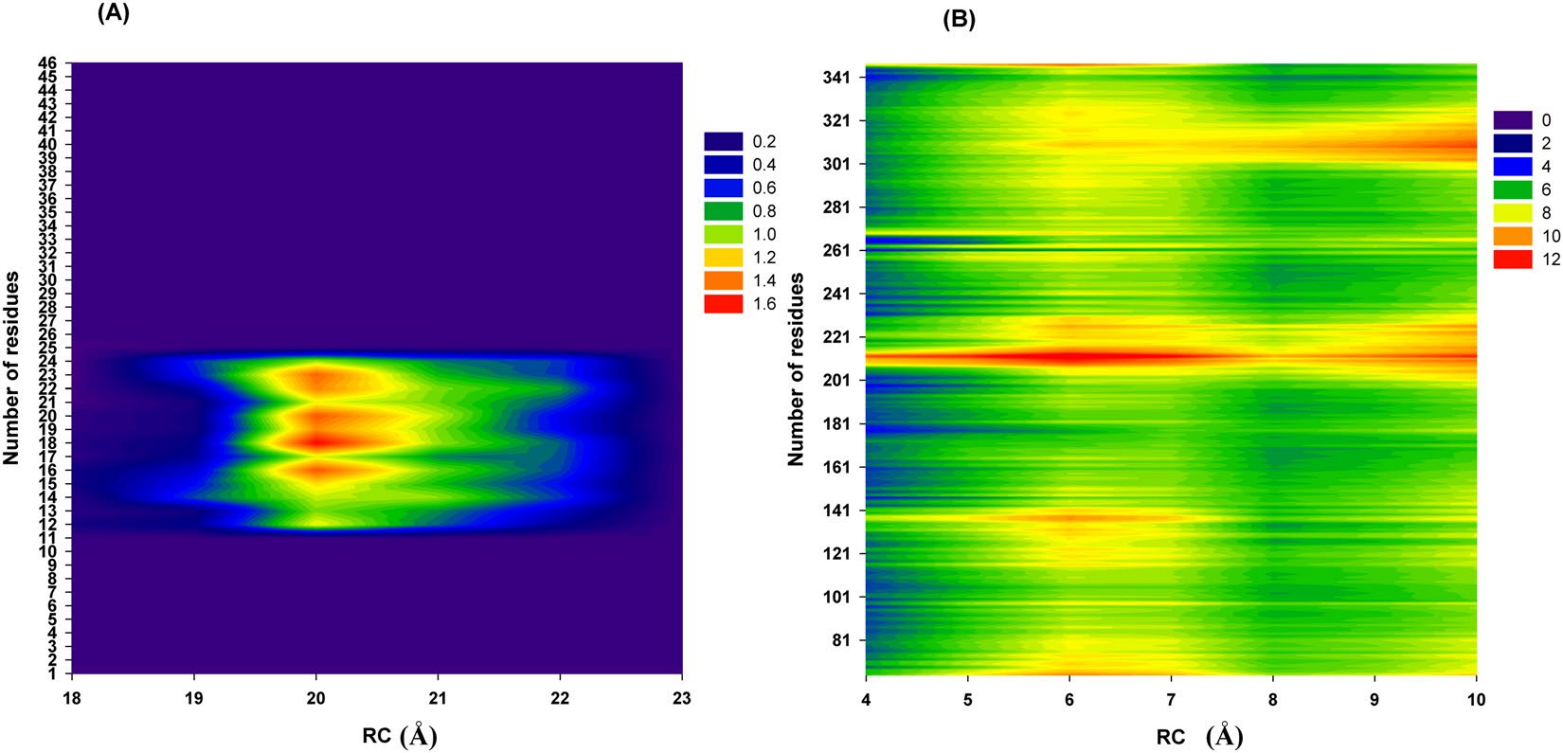
RMSF profiles of MOR with β-funaltrexamine. (**A**) RC: 18–23 Å; the order of the residues is as follows: Met65, Val66, Thr67, Ala68, Ile69, Thr70, Ile71, Met72, Ala73, Leu74, Tyr75, Phe123, Gln124, Ser125, Val126, Asn127, Tyr128, Leu129, Met130, Gly131, Thr132, Trp133, Pro134, Phe135, Lys209, Arg211, Gln212, Gly213, Ser214, Ile215, Asp216, Cys217, Thr218, Tyr299, Lys303, Ile308, Glu310, Thr312, Gln314, Thr315, Val316, Trp318, His319, Phe320, Ile322, and Ala323. (**B**) RC: 3–10 Å.

We also observed in our PMF profiles that β-funaltrexamine must overcome the two energy barriers (major barrier: at RCs of 4–14 Å; minor barrier: RC at 18–23 Å) to bind with the binding pocket (Table 2) of MORs. The possible residues interacting with β-funaltrexamine in the minor barrier (at RCs of 18–23 Å) are shown Figs 1 and 4 as well as Table 1. First, β-Funaltrexamine must overcome the minor energy barrier (2.07 ± 0.45 kcal/mol). At RCs of 19–21 Å, the Phe123, Gln124, Ser125, Val126, Asn127, Tyr128, Leu129, Met130, Gly131, Thr132, Trp133, and Pro134 residues (order: 1–24; RMSF > 1.00 Å) exhibited obvious fluctuations, particularly the Asn127, Leu129, Gly131, and Trp133 residues (order: 16, 18, 20, and 22). Table 2 also showed that the binding interactions were quickly decayed within the RCs of 19–21 Å. Our results showed that the residues might play important roles in relaxing Mors and making β-funaltrexamine easy to overcome the minor energy barrier. Subsequently, β-funaltrexamine must overcome the major energy barrier (6.46 ± 1.06 kcal/mol). At RCs of 7–11 Å, all residues exhibited obvious fluctuations (RMSF > 6 Å). At 4–7 Å, the Met65–Phe87, Leu116–Ser145, Pro201–Asn230, and Ile301–Cys330 residues exhibited obvious fluctuations (RMSF > 8 Å). Table 2 showed that the binding interactions were quickly decayed within the RCs of 4–7 Å. Our results showed that Met65–Phe87, Leu116–Ser145, Pro201–Asn230, and Ile301–Cys330 residues might play important roles in relaxing Mors and making BU72 easy to overcome the major energy barrier.

### Comparing specific changes of the snapshots of RC at 28 Å and X-ray MORs (RC at 3 Å)

For the binding modes of MORs with BU72 and β-funaltrexamine, our results were presented in Figure S5. The binding modes were quite different in the MORs of RC at 3 and 28 Å. The 2000 ns GaMD simulations were performed for the BPSA analyses by using Hollow and UCSF chimera software^34, 35^. The BPSAs of the four MOR conformations (MORs with BU72 at RC of 3 and 28 Å; MOR with β-funaltrexamine at RC of 3 and 28 Å) were 37687.69 (2562 oxygen atoms), 29361.68 (1996 oxygen atoms), 51941.93 (3531 oxygen atoms) and 33877.73 (2303 oxygen atoms) Å^3^, respectively (Figure S6). Our BPSA calculations indicated that the BPSAs of MORs at 28 Å declined sharply.

At RCs of 18–23 Å, our predicted binding mechanisms showed that no residues interacted with BU72 and β-funaltrexamine, and nine residues (Val126, Asn127, Tyr128, Leu129, Met130, Gly131, Thr132, Trp133, and Pro134) exhibited obvious fluctuations and enabled the two compounds to bind to MORs. At RCs of 4–11 Å, our predicted binding mechanisms revealed that 34 residues (Lys209–Phe221 and Ile301–Cys321) exhibited obvious fluctuations and enabled the two compounds to bind with MORs. Figure S4 illustrates the side and top views of the 34 residues (Lys209–Phe221 and Ile301–Cys321) among the four MORs structures (snapshots at an RC of 28 Å: MOR with BU72 and MOR with β-funaltrexamine; X-ray MOR at 3 Å: MOR with BU72 and MOR with β-funaltrexamine). Our results indicated that the 34 residues (Lys209–Phe221 and Ile301–Cys321) were the key regions enabling the two compounds to bind to the active site of the MORs. Our results indicated that the 34 residues (Lys209–Phe221 and Ile301–Cys321) were the key regions enabling the two compounds to bind to the active site of the MORs. When the MORs were in the holo form, the key region was in the open conformation (Figure S4, red part) and the BPSAs declined sharply (Figure S6). When the MORs were in the apo form, the key region was in the closed conformation (Figure S4, green part) and the BPSAs declined sharply (Figure S6).

### Comparing specific changes of the snapshots of RC at 4 and 18 Å

The snapshots of RC at 4 and 18 Å were performed for the BPSA analyses by using Hollow and UCSF chimera software^34, 35^. The BPSAs of the four MOR conformations (MORs with BU72 at RC of 4 and 18 Å; MOR with β-funaltrexamine at RC of 4 and 18 Å) were 35495.86 (2413 oxygen atoms), 31833.01 (2164 oxygen atoms), 50176.7 (3411 oxygen atoms) and 3701.02 (2561 oxygen atoms) Å^3^, respectively.

### Comparing the alternative models describing the transition between active and inactive states in GPCRs

Although Prof. Michel Bouvier proposes the hypothesis of the alternative models describing the transition between active and inactive states in GPCRs (Figure S7)^8^, there is no experimental structural evidence of the ligand-free state in the mu opioid receptor. But, there is a few evidence for ligand-free state of GPCR, such as β1 adrenergic receptor. For the β1 adrenergic receptor, the experimental data (Table S1 and Figure S8) reported by Dr. Huang *et al*. show that the ligand-free state is similar with active state, and the data also support the two-state model illustrated in Figure S7(B) ^36^. Comparing the B1AR (Figure S8) and MOR (Figures S3 and S4), the obvious conformational changes occur in TM1 and TM6, respectively. Dr. Miao *et al*. used the GaMD to study the activation of M2 muscarinic GPCR^37^. The GaMD method may be suitable for studying the activation of GPCRs with agonist and antagonist. Thus, we used the GaMD to study the binding mechanism of MORs with agonist and antagonist. Finally, our GaMD simulation results tended to Figure S7(A).

## Conclusions

In this study, we used GaMD simulations and X-ray structures (agonist and antagonist ligands bound to MORs) to identify the binding mechanisms of MORs to BU72 and β-funaltrexamine. From the X-ray structures, the RCs were defined as the distance between the CM of the compounds and the CM of their binding pockets. Subsequently, we applied the GaMD enhanced sampling method and performed RMSF and PMF calculations to predict the binding mechanisms of the two compounds to MORs. Our PMF calculations indicate the presence of two energy barriers (major barrier: at RCs of 4–14 Å; minor barrier: at RCs of 18–23 Å) in 1000-ns GaMD simulations. For the MOR with agonist (BU72), the major energy barrier was 6.43 kcal/mol and the minor barrier was 1.14 kcal/mol. For the MOR with antagonist (β-funaltrexamine), the major energy barrier was 5.87 kcal/mol and the minor barrier was 1.19 kcal/mol. According to our RMSF profiles, the 34 MOR residues (Lys209–Phe221 and Ile301–Cys321) were the key regions enabling the two compounds to bind to the active site of the MORs. Our results indicated that the 34 residues (Lys209–Phe221 and Ile301–Cys321) were the key regions enabling the two compounds to bind to the active site of the MORs. When the MORs were in the holo form, the key region was in the open conformation (Figure S5, red part) and the BPSAs were increased (Figure S7). When the MORs were in the apo form, the key region was in the closed conformation (Figure S5, green part) and the BPSAs were decreased (Figure S7). The key region might be responsible for the selectivity of new MOR drugs.

## Method

### Gaussian accelerated molecular dynamics (GaMD)

GaMD is an enhanced conformational sampling method of biomolecules by adding a harmonic boost potential to smoothen the system potential energy surface^32^. when the system potential (V) is lower than a referenced energy (E), a harmonic boost potential (ΔV) is added as:

1 $${\rm{\Delta }}V=\frac{1}{2}K{(E-V)}^{2},if\,V < E$$

where K is a harmonic force constant. The modified system potential (V*) is given by:

2 $${V}^{\ast }=V+\frac{1}{2}K{(E-V)}^{2},if\,V < E\,$$

IF the system potential (V) is great than a referenced energy (E), a harmonic boost potential (ΔV) is equal to zero. Smoothening the potential energy surface for overcoming intermedia energy barriers, the boost potential is to satisfy the following step. There are two potential energy values V1 and V2. If V1 < V2, the biased V1* < V2*. By replacing V* with eq. (2), the relationship will be:

3 $$E < \frac{1}{2}(V1+V2)+\frac{1}{K}$$

Step (1) If V1 < V2, the potential difference on the smoothened energy surface should be smaller than that of the original energy surface. By replacing V* with eq. (2), the relationship will be:

4 $$E > \frac{1}{2}(V1+V2)$$

Step (2) Combing the eq. (3), eq. (4) and the relationship (Vmin ≦ V1 < V2 ≦ Vmax), we can derive:

5 $$Vmax\le E\le Vmin+\frac{1}{K}$$

Step (3) Where Vmin and Vmax are the minimum and maximum potential energies. By eq. (5), we can obtain

6 $$\frac{1}{K}\le \frac{1}{Vmax-Vmin}$$

K constant is defined as

7 $$K=K0(\frac{1}{Vmax-Vmin}),0 < K0\le 1$$

k0 is the magnitude of the applied boost potential.

Step (4) The standard deviation of ΔV must be small enough to ensure accurate reweighting^38^.

8 $${\sigma }_{\Delta V}=\sqrt{{(\frac{\partial \Delta V}{\partial V}|V=Vave)}^{2}{{\sigma }_{V}}^{2}}=K(E-Vave){\sigma }_{V}\le {\sigma }_{0}$$

where the Vave and σV parameters are the average and standard deviation of the potential energies, and σΔV is the standard deviation of ΔV with σ0 as a user-specified upper limitation for accurate reweighting potential energies. Here the standard deviations of the total potential and dihedral potential boosts are equal to 10 kcal/mol in our simulations.

Step (5) Extending the step (2). If E = Vmax, we can drive the eq. (5) and obtain

9 $$K0\le \frac{\sigma 0}{\sigma V}\frac{Vmax-Vmin}{Vmax-Vave}$$

According to eq. (21) and eq. (19), the K0 can be defined as:

10 $$K0=min\{1.0,\frac{\sigma 0}{\sigma V}\frac{Vmax-Vmin}{Vmax-Vave}\}$$

Step (6) Extending the step (2). If E = Vmin + 1/k, we can drive the eq. (8) and obtain

11 $$K0\ge (1-\frac{\sigma 0}{\sigma V})\frac{Vmax-Vmin}{Vmax-Vave}$$

Step (7) GaMD can provide the total potential boost, dihedral potential boost, and the dual potential boost to accelerate the molecular simulations. The boost potential (ΔV) is given as:

12 $${\rm{\Delta }}V=\frac{1}{2}K0\frac{1}{{V}_{max}-{V}_{min}}{(E-V)}^{2},if\,V < E$$

where K0 is the magnitude of the applied boost potential, Vmin and Vmax are the system minimum and maximum potential energies. The initial K0 is equal to 1.0, and the Vamx and Vmin will be obtained form our cMD simulations. To characterize the extent to which ΔV follows Gaussian distribution, its distribution anharmonicity^32^. GaMD method has been applied in the alanine dipeptide, chignolin and lysozyme simulations^32^.

### Reweighted free energy calculations for GaMD simulations (Gaussian Approximation)

The probability distribution of the selected reaction coordinates A(r) is defined as P*(A), where r can be distance, angle, RMSD, etc.^38^. According to the GaMD boost energies of each reaction coordinate, P*(A) can be reweighted and defined as

13 $$P({A}_{j})={P}^{\ast }({A}_{j})\frac{{\langle {e}^{\beta {\rm{\Delta }}V(r)}\rangle }_{j}}{{\sum }_{j=1}^{M}{\langle {e}^{\beta {\rm{\Delta }}V(r)}\rangle }_{j}},J=1\, \sim \,M$$

where M is the number of bins, β is equal to KBT, ${\langle {e}^{\beta \Delta V(r)}\rangle }_{j}$ is the ensemble-average factor of the jth bin. For reducing the energetic noise, the ensemble-average factor can be defined as:

14 $$\langle {e}^{\beta \Delta V(r)}\rangle =\exp \{\sum _{K=1}^{\infty }\frac{{\beta }^{K}}{K!}{C}_{K}\}$$

After driving the eq. (14), the first three cumulants can be defined as:

15 $$\begin{array}{c}C1=\langle {\rm{\Delta }}V\rangle ,\\ C2=\langle {\rm{\Delta }}{V}^{2}\rangle -\,{\langle {\rm{\Delta }}V\rangle }^{2},\\ C3=\langle {\rm{\Delta }}{V}^{3}\rangle -3\langle {\rm{\Delta }}{V}^{2}\rangle \langle {\rm{\Delta }}V\rangle +2{\langle {\rm{\Delta }}V\rangle }^{2}\end{array}$$

The reweighted free energies can be calculated by

16 $$F({A}_{j})=-\frac{1}{\beta }lnP({A}_{j})$$

### GaMD simulation of MORs

**Figure 5:**
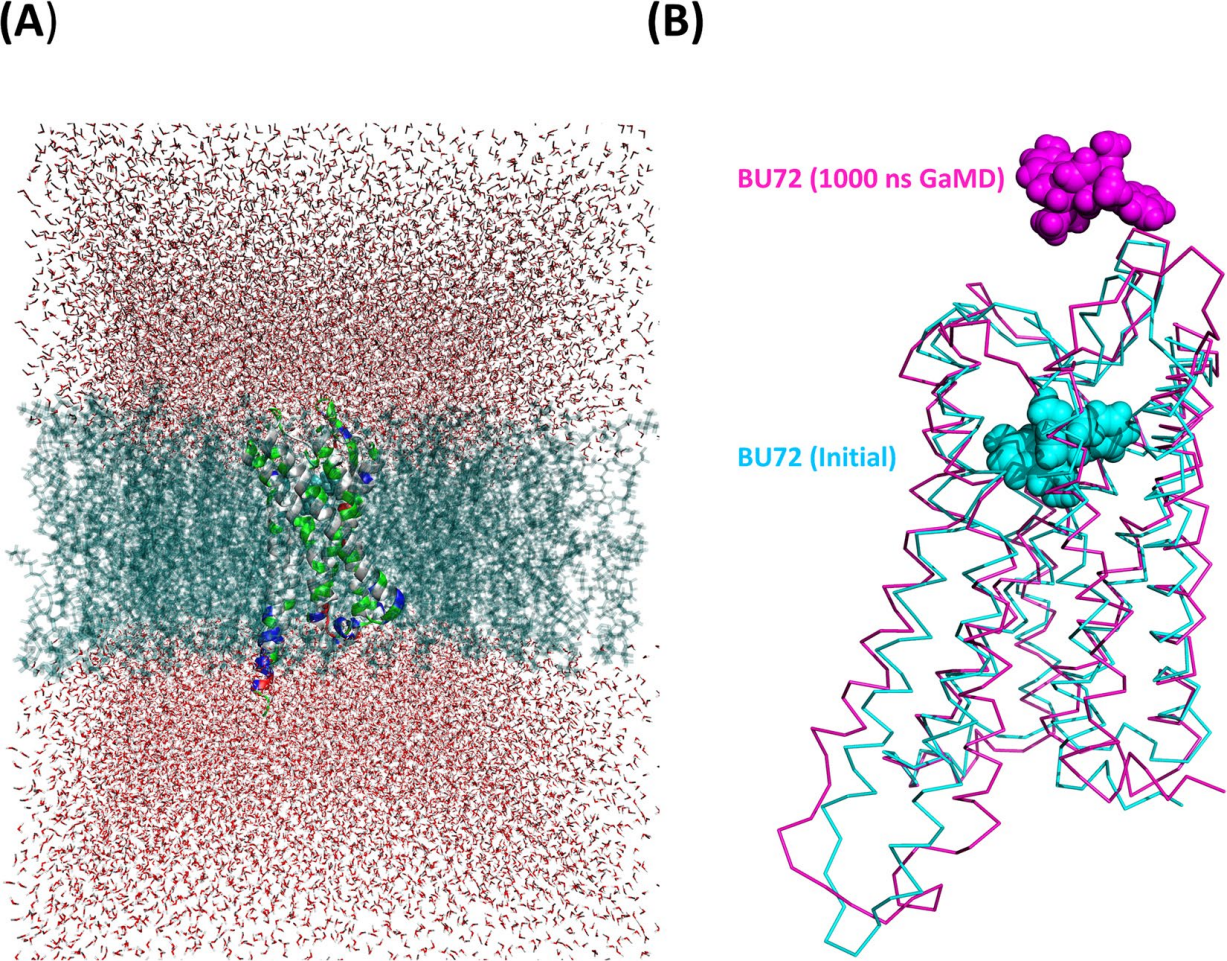
(**A**) Initial structure of an active MOR with BU72. (**B**) Snapshots of an active MOR with BU72 (cyan: initial structure; magenta: structure derived after 1000-ns GaMD).

Firstly, we modified the inactive MOR pdb file, and we used pymol software to break the covalent bond of the antagonist (β-funaltrexamine) with Lys233 residues. Secondly, we generated our initial models (inactive, PDB ID: 4DKL/our modified pdb file; active, PDB ID: 5C1M) by using the CHARMM-gui server^39^. The initial MOR structures were generated and then inserted into solvent molecules. The solvent molecules contained a POPC lipid bilayer with 20% cholesterol, TIP3 water, and 0.15 M NaCl molecules^40, 41^. The size of the MOR system was approximately 11.00 × 11.00 × 14.00 nm^3^. The initial MOR structures were then simulated with the AMBER 14 package by using the AMBER FF14 all-hydrogen amino acid, AMBER lipid 14, and AMBER GAFF force field parameters. The AMBER GAFF partial atomic charges are often based on the RESP fitting procedure of the electrostatic potential obtained at the HF/6–31 G(d) level of theory. The geometries of a morphine agonist (BU72) and antagonist (β-funaltrexamine) were fully optimized, and their electrostatic potentials were obtained using a single-point calculation. Both operations were performed at the HF level with the 6–31 G(d,p) basis set by using the GAMESS US program^42^. All cMD simulations were performed in the isothermal–isobaric ensemble with a simulation temperature of 310 K, unless stated otherwise, by using a Verlet integrator with an integration time step of 0.002 ps and SHAKE constraints^43^ of all covalent bonds involving hydrogen atoms. In the electrostatic interactions, atom-based truncation was performed using the PME method^44^, and the switch van der Waals function was used with a 2.00 nm cutoff for atom-pair lists. The complex structure was minimized for 100,000 conjugate gradient steps and was then subjected to a 100-ns isothermal, constant-volume MD simulation and five independent 1000-ns GaMD simulations. Figure 5A shows the initial structure of the active MOR with UB72.

### Free energy, binding pocket site area, root-mean-square fluctuation, and electrostatic/van der Waals binding interactions calculations (RMSF)

For the active MOR, the reaction coordinates (RCs) were defined as the center of mass distances between BU72 and the binding pocket (Tyr148, Asp147, Ile322, Ile144, Val236, Met151, Val300, Ile296 and Tyr326). For the inactive MOR, the RCs were defined as the center of mass distances between β-funaltrexamine and the binding pocket (Asp147, Tyr148, Lys233, Lys303, Val236, Val300, Met151, Ile296, Ile322, Trp293 and Tyr326). The binding modes of MORs with BU72 and β-funaltrexamine are shown in Fig. 2. Root-mean-square fluctuation (RMSF) calculations were conducted for two barriers (at RCs of 4–10 and 18–23 Å). For MORs with BU72 (at RCs of 18–23 Å), RMSF calculations were performed for the corresponding 32 residues within 10 Å of BU72 (snapshots of MORs with BU72 at an RC of 18 Å); the 32 residues are Val126, Asn127, Tyr128, Leu129, Met130, Gly131, Thr132, Trp133, Pro134, Cys140, Val143, Ile144, Asp147, Lys209, Tyr210, Arg211, Gln212, Gly213, Ser214, Ile215, Asp216, Cys217, Thr218, Leu219, Thr225, Trp226, Glu229, Lys303, Ala304, Leu305, Thr307, and Glu310. For MORs with BU72 (at RCs of 4–10 Å), RMSF calculations were performed for all residues of the MOR. For MORs with β-funaltrexamine (at RCs of 18–23 Å), RMSF calculations were performed for the corresponding 32 residues within 10 Å of β-funaltrexamine (snapshots of MORs with β-funaltrexamine at an RC of 18 Å); the 46 residues are Met65, Val66, Thr67, Ala68, Ile69, Thr70, Ile71, Met72, Ala73, Leu74, Tyr75, Phe123, Gln124, Ser125, Val126, Asn127, Tyr128, Leu129, Met130, Gly131, Thr132, Trp133, Pro134, Phe135, Lys209, Arg211, Gln212, Gly213, Ser214, Ile215, Asp216, Cys217, Thr218, Tyr299, Lys303, Ile308, Glu310, Thr312, Gln314, Thr315, Val316, Trp318, His319, Phe320, Ile322, and Ala323. For MORs with β-funaltrexamine (RC at 4–10 Å), RMSF calculations were performed for all MOR residues. The RC profiles, the intermolecular interaction energy, and RMSF profiles were analyzed using AmberTools 16. The RC profiles were calculated for the RCs of the free energy (or potential of mean force, PMF) calculations. The PyReweighting toolkit^38^ was used to reweight the GaMD simulations for calculating the PMF profiles and to examine the boost potential distributions. One-dimensional PMF profiles were also constructed using RCs for MORs with a bin size of 1.0 Å. For Figs 4–7 the binding pocket site area (BPSA) of MORs was analyzed using Hollow and UCSF chimera software^34, 35^. The electrostatic/van der Waals binding interactions between key residues (Table 1) and the two compounds were conducted for two barriers (at RCs of 4–10 and 18–23 Å), and the binding interactions were carried out with the program sietraj^45^.

## Electronic supplementary material

SUPPLEMENTARY INFO
