# Supplementary material for: Understanding the molecular basis of agonist/antagonist mechanism of human mu opioid receptor through gaussian accelerated molecular dynamics method
Source: Sci Rep. 2017 Aug 10;7:7828. doi: 10.1038/s41598-017-08224-2 (PMC5552784; doi:10.1038/s41598-017-08224-2)
Supplement: Supplementary file 1 — SUPPLEMENTARY INFO [file 41598_2017_8224_MOESM1_ESM.doc]

**Understanding the molecular basis of agonist/antagonist mechanism of human mu opioid receptor through gaussian accelerated molecular dynamics method**

Yeng-Tseng Wang^abcd*^ and Yang-Hsiang Chan^e^

^a^.Department of Biochemistry, College of Medicine, Kaohsiung Medical University, Taiwan

^b.^Center for Biomarkers and Biotech Drugs, Kaohsiung Medical University, Kaohsiung, Taiwan

^c.^Graduate Institute of Medicine, Kaohsiung Medical University, Kaohsiung, Taiwan

^d^Department of Medical Research, Kaohsiung Medical University Hospital, Kaohsiung, Taiwan

^e^Department of Chemistry, National Sun Yat-sen University, 70 Lien Hai Road, Kaohsiung, Taiwan

*Correspondence: E-mail: [c00jsw00@kmu.edu.tw](mailto:c00jsw00@kmu.edu.tw) or c00jsw00@gmail.com (Yeng-Tseng Wang)

**
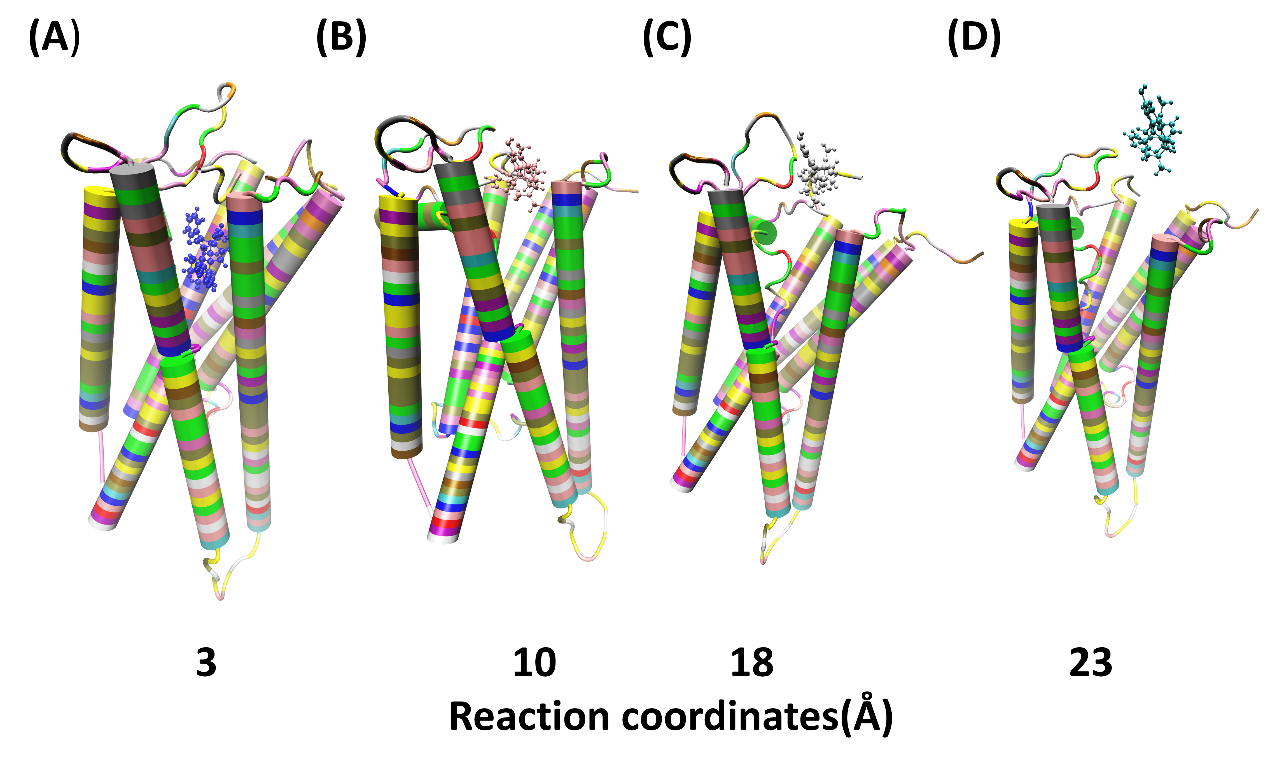
**

**Figure S1.** Snapshots of an MOR with BU72 at reaction coordinates of (A) 3, (B) 10, (C) 18, and (D) 23 Å.


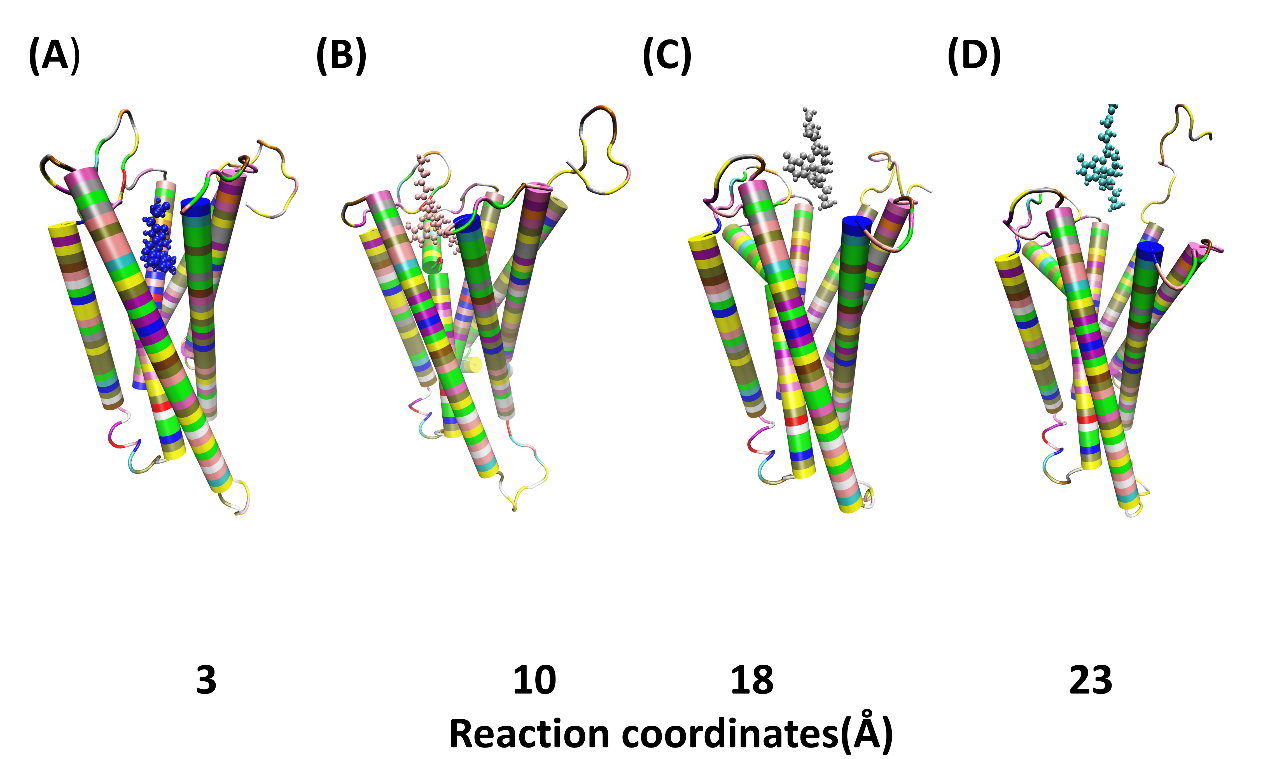


**Figure S2.** Snapshots of an MOR with β-funaltrexamine at reaction coordinates of (A) 3, (B) 10, (C) 18, and (D) 23 Å.

**
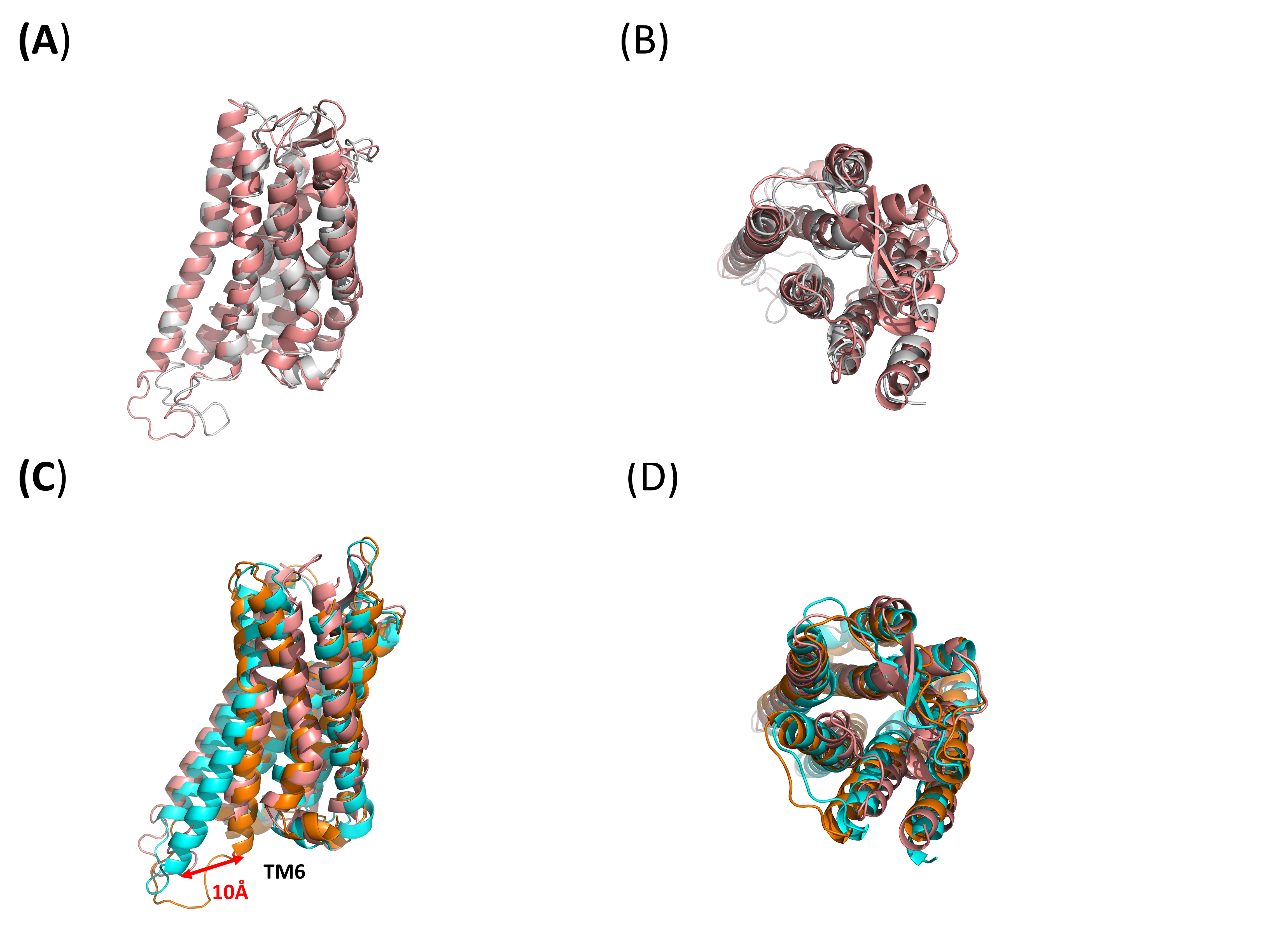
**

**Figure S3.** (A) Snapshots at a reaction coordinate (RC) of 28 Å (wheat: MOR with BU72; white: MOR with β-funaltrexamine). (B) Top view of (A). (C) Comparison of the X-ray MOR structures with the snapshots at an RC of 28 Å (cyan: X-ray structure of the MOR with BU72; orange: X-ray structure of the MOR with β-funaltrexamine; wheat: the MOR with BU72 at an RC of 28 Å). (D) Top view of (C).


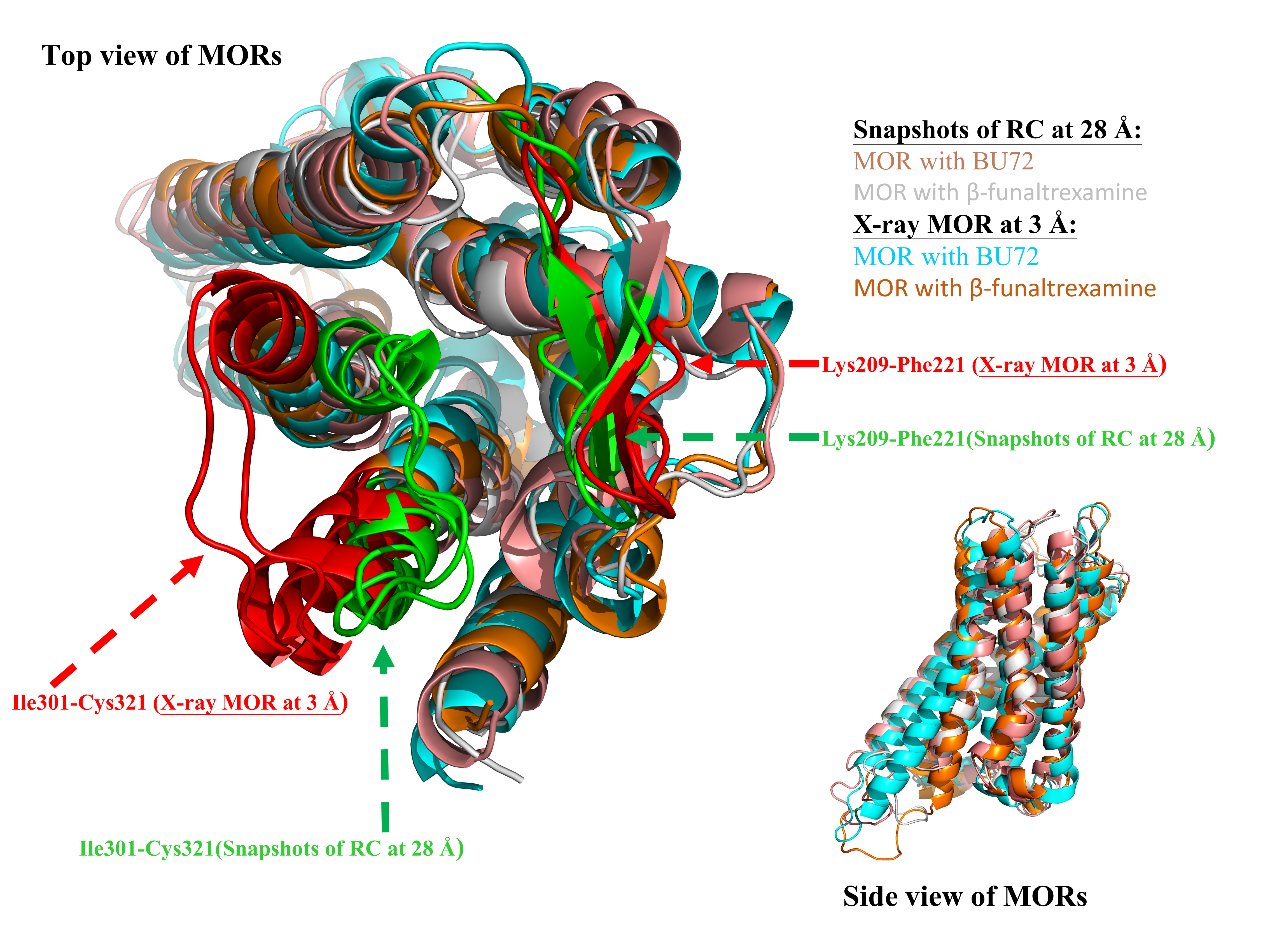


**Figure S4.** Side and top views of the 34 residues (Lys209–Phe221 and Ile301–Cys321) of the MORs.


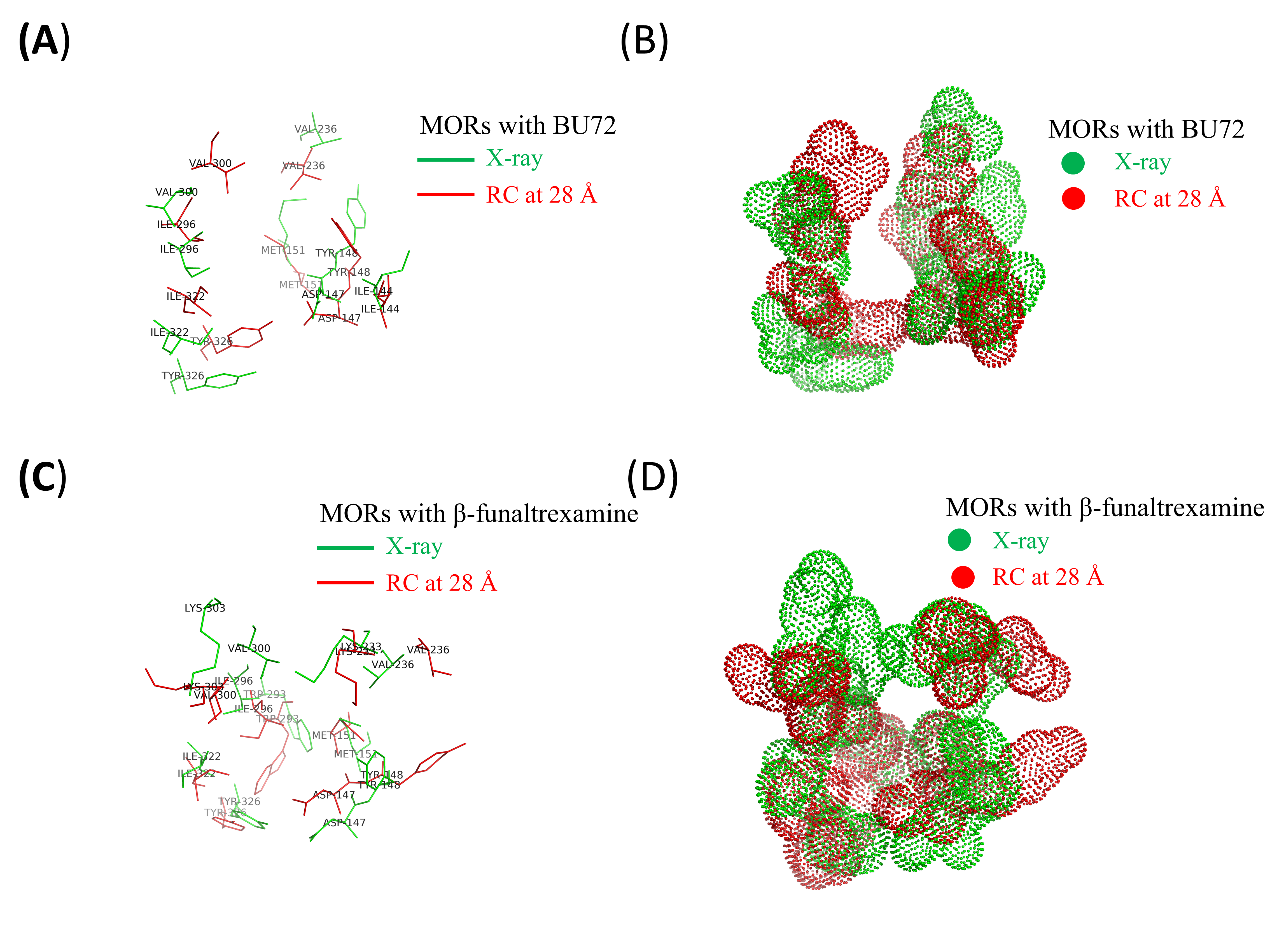


**Figure S5.** The binding modes analysis of MORs with BU72 and β-funaltrexamine.


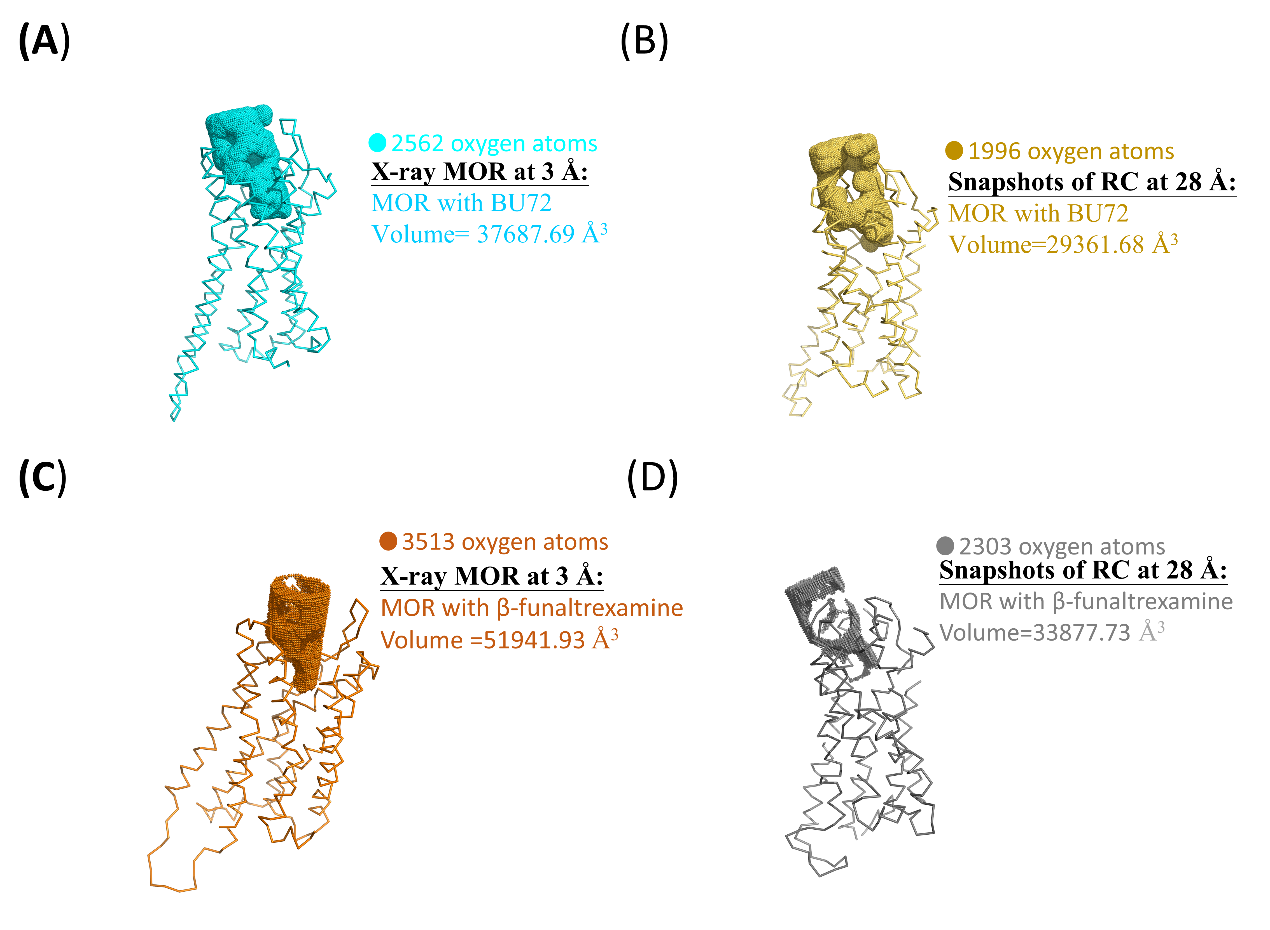


**Figure S6.** The binding pocket site area analysis of MORs with BU72: Snapshots at a reaction coordinate (RC) of : (A) 3 Å and (B) 28 Å. The binding pocket site area analysis of MORs with β-funaltrexamine: Snapshots at a reaction coordinate (RC) of: (C) 3 Å and (D) 28 Å.


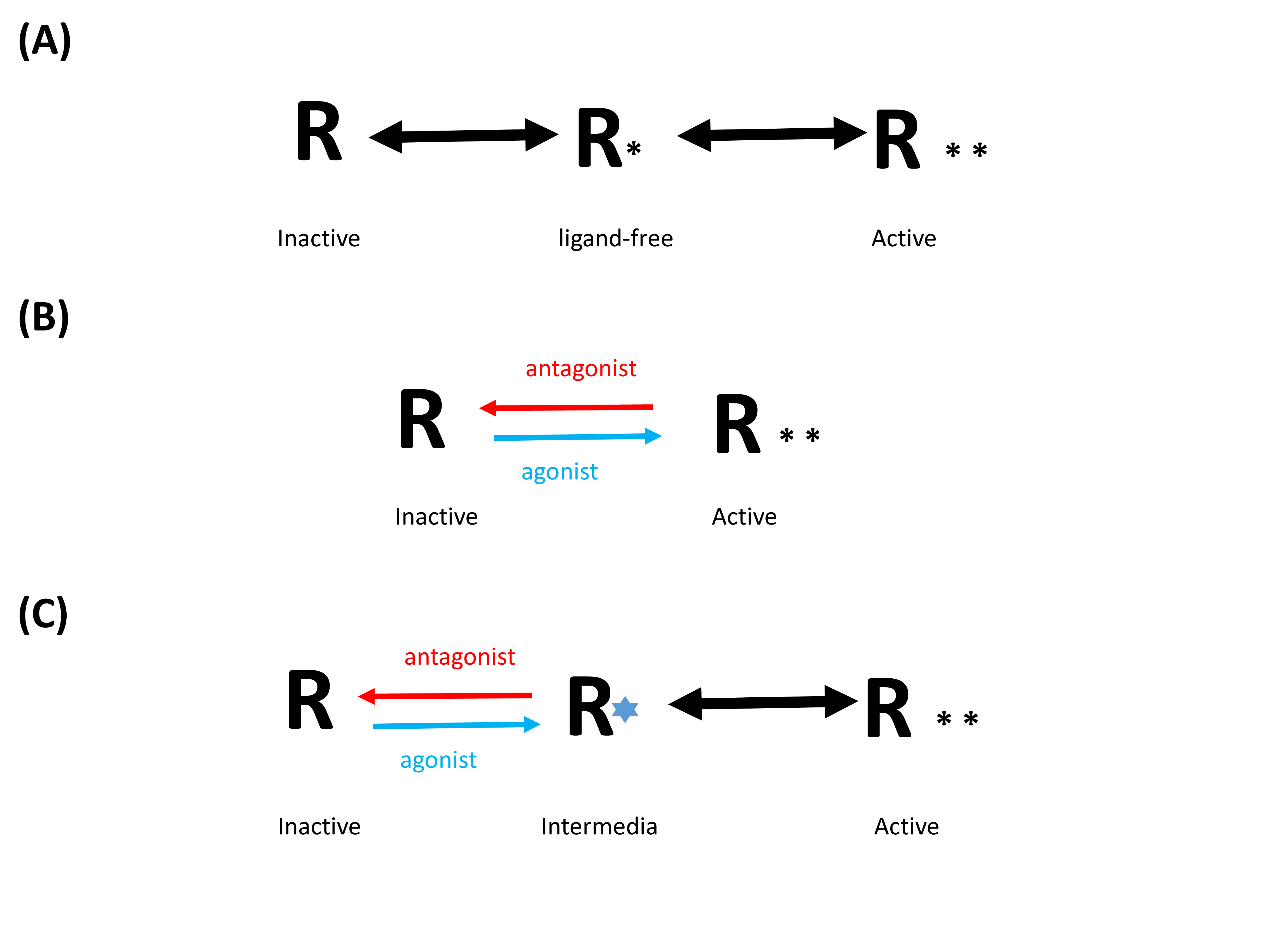


**Figure S7.** Alternative models describing the transition between active and inactive states in GPCRs .(doi:10.1038/nsmb.2584 )


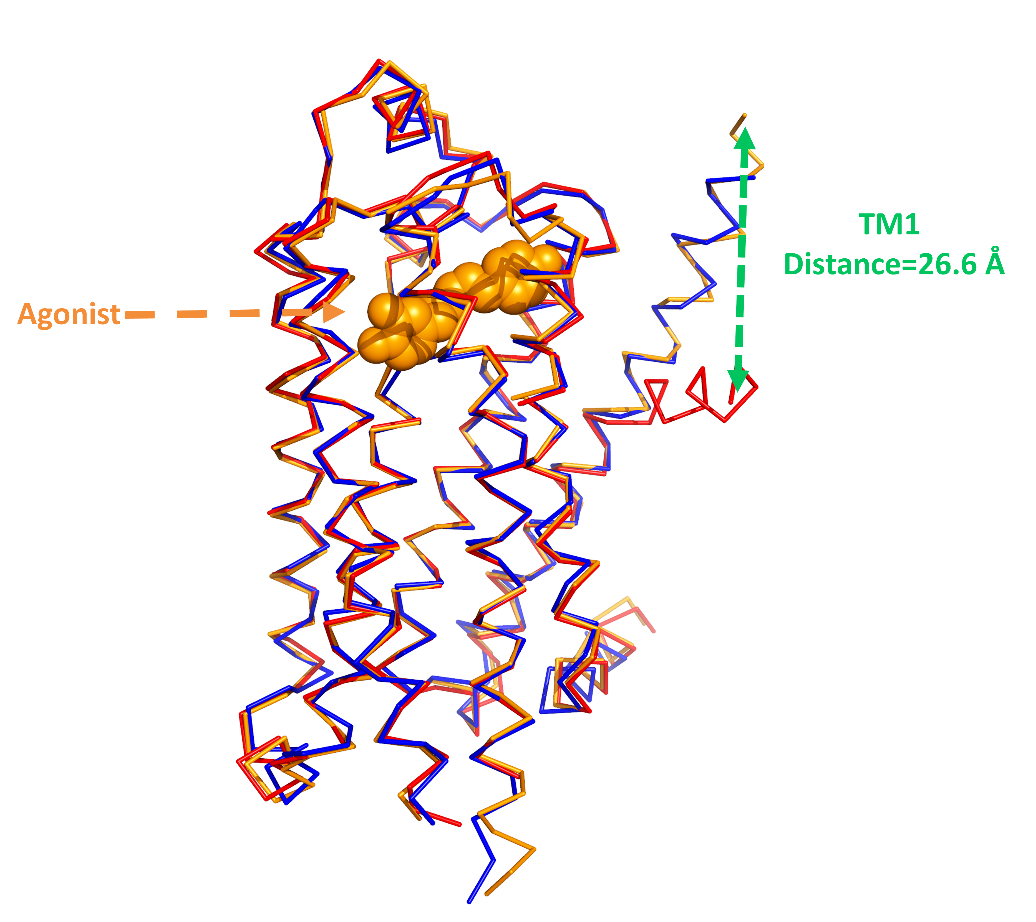


**Figure S8.** Comparison of the X-ray β1 adrenergic receptor structures (orange: active state; blue: ligand-free state; red: inactive state)

**Table S1**. The experimental structure data of GPCR (PDB ID)

| Kind of GPCR | Active state | intermedia state | Inactive state |
| --- | --- | --- | --- |
| β1 adrenergic receptor (B1AR) | 2y00 | 4gpo | 2vt4 |
| mu opioid receptor (MOR) | 5c1m | null | 4kdl |
